# Physical Activity in 3–6 Year Old Children Measured by SenseWear Pro®: Direct Accelerometry in the Course of the Week and Relation to Weight Status, Media Consumption, and Socioeconomic Factors

**DOI:** 10.1371/journal.pone.0060619

**Published:** 2013-04-03

**Authors:** Yvonne Vorwerg, David Petroff, Wieland Kiess, Susann Blüher

**Affiliations:** 1 Integrated Research and Treatment Center (IFB) Adiposity Diseases, University of Leipzig, Leipzig, Germany; 2 Department of Women and Child Health, Hospital for Children and Adolescents, University Hospital of Leipzig, Leipzig, Germany; 3 Clinical Trial Centre, University of Leipzig, Leipzig, Germany; Aga Khan University, Pakistan

## Abstract

**Background:**

Data on objectively measured physical activity (PA) in preschoolers are controversial. Direct accelerometry was performed in children aged 3–6 years, and differences in PA patterns over the course of the week were evaluated. Data were analyzed with gender, BMI, lifestyle, and socioeconomic parameters as covariates.

**Methods:**

PA was measured in 119 children by the SensewearPro® accelerometer and analyzed in the 92 (40 girls) that wore it for at least 4 days including one day of the weekend. Median measuring time in this group was 7 consecutive days (median/mean daily measuring time: 23.5 h/d and 21.8 h/d, respectively), corresponding to 834,000 analyzed minutes. PA questionnaires were completed by 103 parents and 87 preschool teachers to collect anthropometric, lifestyle, and socioeconomic data.

**Results:**

Median daily PA (MET>3) was 4.3 hours (mean: 4.4 hours). Boys spent an estimated 52 min/week more being very active (MET>6) than girls (95% CI [6, 96] min/week, p = 0.02). PA was lower during the weekend (3.7 h/d) compared to weekdays (4.5 h/d), p = 3×10^−6^), where a 95% CI for the difference is [0.5, 1.0] h/d. PA levels did not differ between overweight/obese children (median 4.7 h/d) and normal-weight peers (median 4.2 h/d). Daily media consumption increased with decreasing social class on weekdays (p = 0.05) and during the weekend (p = 0.01), but was not related to the amount of daily PA. A multivariate regression with BMI-SDS as independent variable and gender, age, amount of PA>6 MET, parental BMI, media time and socioeconomic status as explanatory variables revealed that only SES had a significant contribution.

**Conclusion:**

The negative impact of obesity-promoting factors in older children is rather low for preschoolers, but there is evidently a gradient in PA between weekdays and weekends already in this age group. Weight status of preschoolers is already considerably influenced by SES, but not physical activity levels.

## Introduction

Objectively measured physical activity (PA) is inversely associated with markers of insulin resistance, hyperlipidemia, arterial hypertension, and clustered metabolic risk even in children. These associations are partly independent of weight status and are also observed in healthy, normal weight children. Regular PA is essential for maintaining muscular function, metabolic homeostasis and endocrinological as well as immunological health [1]–[3]. In contrast, physical inactivity seems to promote the development of obesity and profound sequelae already at a young age, and this fact is of special importance since physical performance/PA in children has declined significantly over the past decades [4]. The prevalence of childhood overweight and obesity is at an alarmingly high level, and the development of overweight and obesity is closely associated with lack of PA [5], [6]. Physical inactivity seems to track from childhood into adulthood, and up to 80% of overweight or obese children remain obese in adulthood with significant impact on adult health [7]. However, obesity and associated sequelae such as type 2 diabetes are preventable: Following relatively modest changes in lifestyle patterns that include healthy eating patterns and increasing physical activity can reduce the risk of developing diabetes by over 50% in subjects with impaired glucose tolerance. Thus, a comprehensive approach to obesity and diabetes prevention has been suggested that combines population based primary prevention programs. The use of accelerometers in the prevention of chronic disease has also been recommended [8].Thus, objectively measured levels of PA and promotion of PA in childhood may have long-term implications for interventions aimed at improving physical fitness and preventing obesity later in life. Additional factors that are closely involved in the development of childhood obesity and should thus be taken into consideration include socioeconomic status [9] and a sedentary lifestyle. A significant influence on children's day-to-day activity levels seems to be provided by the “shared environment” or specific environmental characteristics, such as preschool or school environments [10].

Accelerometry is regarded the “gold standard” for assessing objectively measured PA, as it provides a reliable and valid estimate of energy expenditure as well as the amount of time spent in sedentary/light PA, moderate PA, and vigorous PA [11], [12]. PA seems to decline starting at school age, with children spending approximately 10% less time in physical activity for each advancing year of age [13]. Thus, preschool age is regarded as a critical period to intervene and to promote physical activity [14]. However, a prerequisite is to understand the physical (in)activity level of preschool-aged children. Valid data on PA levels in children of this age group, covering more than 4 days in order to identify patterns of children's PA between days, are limited [3], [12]. In addition, daily PA seems to be lower during the weekend compared to weekdays in older children [3], [15], [16]. The aim of this study was to evaluate objectively measured PA in 3–6 year old children to examine differences in PA patterns among weekdays and weekends. Associations were also analyzed between PA data and gender, Body-Mass-Index (BMI) as marker of weight status, lifestyle, and socioeconomic parameters.

## Methods

### Subjects

PA was measured in a total of 119 children aged 3–6 years attending preschools by direct accelerometry. Data from 92 children who wore the device for at least 4 days including one day of the weekend, from 103 parents (16 parents did not complete the questionnaires), and from 87 preschool teachers were utilized for the analyses.

### Ethics Statement

The study has been approved by the Institutional Review Board of the University of Leipzig. Written informed consent was obtained by all parents prior to the study.

### Measurements

#### Anthropometry

Height was measured using a digital statiometer (“Dr. Keller III”, Günter GmbH, Tauscha, Germany; precision±2 mm), and weight was determined using a digital scale, with children only wearing light underwear (SECA^®^-scale, Vogel & Halke GmbH, Hamburg, Germany; precision±100 g). Body mass index (BMI) was calculated and was interpreted according to German reference BMI percentiles [17]. Overweight was defined as BMI >90^th^ percentile, and obesity was defined as BMI above the 97^th^ percentile. Normal weight was defined as BMI between the 10^th^ and the 90^th^ percentiles [17].

#### Assessment of physical activity

Accelerometers have been shown to provide valid and reliable assessments of PA in preschoolers-aged children, and direct accelerometry can be appropriately used as a measure of PA in this age group [11], [18]. Especially triaxial accelerometers are valid devices with similar classification accuracy for sedentary, light, and moderate-to-vigorous levels of PA in preschoolers [19]. However, in order to obtain reliable data, a minimum amount of measured days/daily hours of measurement should be obtained, and at least one weekend day should be included in analyses to reliably estimate physical activity levels for preschool children [20]. In our study, PA was measured by accelerometers (SenseWear Pro 2, Bodymedia, SMT medical GmbH&Co Würzburg, Germany [21], [22] over a period of 7 consecutive days, thereby including one weekend. The device acquires and records data from four fundamental sensors: skin temperature, heat flux, galvanic skin response, and tri-axial acceleration (i.e. movement) [22]. The intensity of PA was categorized as *metabolic equivalent* (MET), a physiological concept expressing the energy cost of physical activities as multiples of the resting metabolic rate. MET is defined as the ratio of the metabolic rate (i.e., the rate of energy consumption) during a specific physical activity to a reference rate of the metabolic rate at rest, set by convention to 3.5 mL O_2_·kg^−1^·min^−1^ or equivalently 1 kcal·kg^−1^·h^−1^. A measurement of 1 MET is considered to be the resting metabolic rate obtained during quiet sitting. Four categories with age-specific count cutoffs were analyzed: sedentary activity (MET ≤ 1.4), light physical activity (1.5–2.9 METs), moderate physical activity (3–5.9 METs), and vigorous physical activity (>6 METs) [16], [23]. Sensitivity and specificity for accelerometer-defined boundaries have been shown to be high for sedentary and vigorous physical activity and still acceptable for light and moderate physical activity in young children [24].

### Questionnaires

Data on length and type of PA, environmental factors, family status, number of hours of media consumption and social factors were assessed by parental questionnaires used in the national KIGGS survey [25]. With regard to daily media consumption, parents were given the option of saying that the amount of TV their child watched daily was a) never, b) roughly 30 minutes, c) 1–2 hours, d) 3–4 hours, e) more than 4 hours. Social status was evaluated based on parental education, occupation and household net income [9]. Preschool teachers were also asked to complete a questionnaire to evaluate the daily PA of participating children, including information on playing habits, length and type of PA, behavioral patterns and parental influence. They were also asked to rate the child's favorite activities on an 11 point scale ranging from “coloring and arts and crafts” (0 points) to “playing centering on physical activity” (10 points).

To prove whether the accelerometers are accepted by children of this age group and whether the study design and the applied questionnaires are feasible, 20 preschool-aged children were acquired to wear the accelerometer for one complete day (from 9 am to 9 am). Out of these 20 children, 18 children wore the accelerometer for at least 90% of the time. The questionnaires were tested by their parents and preschool teachers. Interviews with parents and teachers revealed that the acceptance of the accelerometers by the children was good, and that parents and preschool teachers had no difficulties with the study design and with completing the questionnaires. Participating children of the pilot phase were not included in the study population. After the feasibility check, the questionnaire was handed out to all participating parents and preschool teachers and was completed in parallel with the assessment of direct accelerometry.

### Statistical analyses

Data are described by median, interquartile range (IQR), mean and standard deviation (SD). To test for differences in a parameter between groups, a Wilcoxon test is used. In assessing the relation between a continuous and a semi-discrete variable (such as a scale in a questionnaire running from 1 to 10), a Spearman rank correlation was used. The differences in proportions between two groups were compared with a chi-squared test and for an ordered set of more than two groups, a test for trends in proportions was used. A multivariate analysis of terms potentially affecting childrens' weight status was included and described below. A p-value ≤0.05 was considered to be significant. Analyses were carried out using R version 2.11.1 [26] and PASW 18 (IBM SPSS Statistics 18).

## Results

### Response rates

A total of 1053 families from 13 different preschools were asked to participate. Of these, 119 children from 8 preschools participated in this study (66 boys). One major problem in recruiting children in this age group was that many of the parents would not allow their children to wear the accelerometers over a consecutive period of 7 days. In addition, the proposed duration of 7 consecutive days of measurement could only be reached by 92 out of the 119 participating children. Reasons for wearing the device for less than 4 days, as indicated by parents, included: discomfort with the device, technical problems with the accelerometer, device was worn only during days but not during nights, acute illness, spontaneous vacation, red spots at the wearing site and refusal to wear the device for more than 4 days by children. These children were excluded from further analyses. Thus, measurements from 92 children (40 girls) who wore the device for at least 4 consecutive days and nights, including one day of the weekend, were further evaluated. Within these 92 children, median measuring time was 7 consecutive days (median/mean daily measuring time: 23.5 h/d and 21.8 h/d, respectively), corresponding to 834,000 analyzed minutes. Out of the 119 participating families, questionnaires of 103 parents were returned and could be used for the analyses.

### Anthropometric characteristics

The median age of the study cohort was 5.3 years for boys and 5.0 years for girls. Median BMI-SDS was 0.26 for boys and 0.42 for girls. Detailed baseline characteristics of the study group are provided in Table 1. The number of overweight and obese children in the study population (BMI > 90^th^ percentile) was 13 (14%). Out of them, 8 children were overweight (BMI 90^th^–97^th^ percentile), and 5 children were obese (BMI >97^th^ percentile). 7 children had a BMI between the 3^rd^ and 10^th^ percentile (threshold of being underweight). For further analyses, overweight and obese children were combined into one study group (ov/ob), and all children with a BMI between the 3^rd^ and the 90^th^ percentile were regarded as one group.

**Table 1:** Baseline characteristics of study population used for PA analyses.

		Age (years)	Height (cm)	Weight (kg)	BMI- SDS	Measuring time (h)
**Boys** N = 52	median	5.3	115	19.6	0.26	23.5
	IQR	[4.6, 6.0]	[106.8, 117.0]	[17.3, 21.6]	[−0.37, 0.80]	[22.3, 23.9]
	mean	5.3	112.4	20.1	0.08	21.7
	SD	0.9	6.7	3.7	1.1	4.1
**Girls** N = 40	median	5.0	109	18.3	0.42	23.6
	IQR	[4.4, 5.7]	[105.8, 115.5]	[17.4, 23.0]	[−0.26, 1.27]	[22.5, 24.0]
	mean	5.3	110.1	20.0	0.42	21.8
	SD	0.9	6.9	3.9	1.0	4.3
p-value Wilcoxon test (boys vs.girls)		0.3	0.1	1.0	0.2	0.7

### Socioeconomic characterization

Out of the 103 parents who returned the questionnaires, complete data on parental education, occupation and household-net-income to calculate the social status were obtained from 83 parents. Out of them, 21 families belonged to the lower social class, 42 to the middle social class, and 20 to the upper social class, as defined by Winkler [9]. 20 participating families could not be classified, as data were incomplete.

With increasing social status, the number of ov/ob children significantly declined: The ratio of ov/ob children to the remaining children was highest in families with low social status (7∶14), in the middle for families with an intermediate social status (4∶38) and was lowest in families with high social status (1∶19), (p = 0.009).

### Physical activity

#### Daily physical activity

Direct accelerometry was assessed based on PA data obtained from accelerometers *SenseWear Pro®*. Median daily PA (MET>3) of all investigated children was 4.3 hours (IQR [3.1, 5.5] hours) and the mean was 4.4 hours (SD 1.8 hours). The suggested minimum amount of 60 minutes per day for this age group spent at moderate-to-vigorous physical activity was achieved by all children participating in our study when averaging their weekly values.

#### Gender differences in PA in preschoolers-aged children

**Figure 1:**
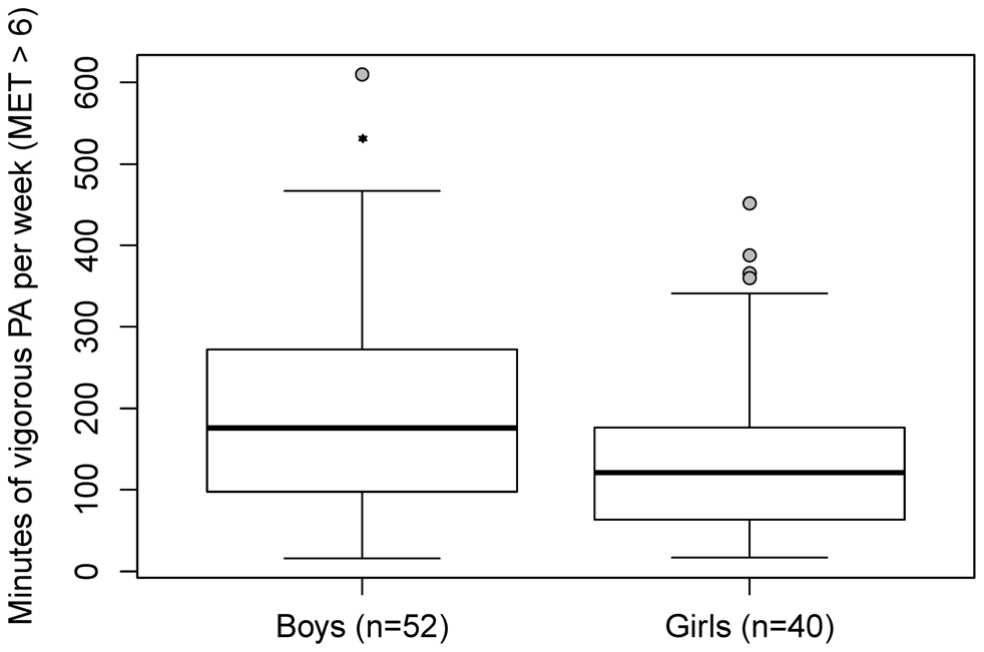
Gender differences in weekly vigorous physical activity (amount of time being very active, MET>6) in preschoolers children aged 3–6 years (*: p = 0.02).

There were significant differences in weekly PA (amount of time being very active, MET>6) in children aged 3–6 years between girls and boys: Boys spent an estimated 52 min/week (95% CI [6, 96] min/week, p = 0.02) more performing vigorous activities (MET>6) than girls (boys: median 176 min, mean 210 min; girls; median 121 min, mean 150 min) (Fig. 1). However, total PA (MET>3) did not depend significantly on gender (boys: median PA 4.4 h/d, IQR [3.2, 5.5], mean PA 4.4 h/d, SD 1.7; girls: median PA 4.1 h/d, IQR [3.0, 5.6], mean PA 4.4 h/d, SD 2.0, p = 0.5).

#### Differences in physical activity between weekdays and weekends

**Figure 2:**
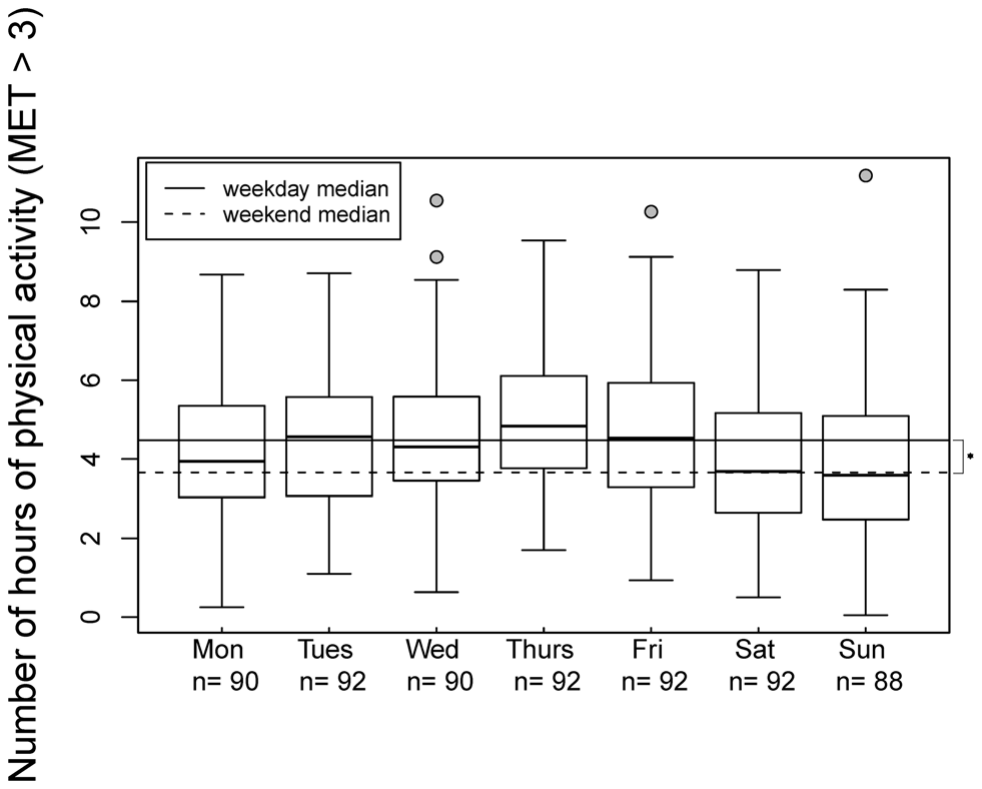
Daily hours of total physical activity (MET>3) in preschool children aged 3–6 years over the course of the week. (Median on weekends 3.7 h/d and on weekdays 4.5 h/d; *: p = 3×10^−6^).

Analysis of PA over the course of the week showed that PA was considerably and significantly lower during the weekend compared to weekdays (weekend median: 3.7 h/d, weekday median: 4.5 h/d, 95% CI for location shift = [0.5, 1.0] h/d, p = 3×10^−6^). As can be seen in Fig. 2, the median on any given weekday was higher than that on the weekend. The magnitude of the difference between weekdays and the weekend was estimated to be similar over all social classes, though the number of data available was too small to show the difference significantly for the lower and upper social classes alone: the location shift was 0.8 h/d for the lower class (95% CI = [0, 1.5] h/d, p = 0.07, n = 14), 0.5 h/d for the middle class (95% CI = [0.2, 0.9] h/d, p = 0.004, n = 36), and 0.7 h/d for the upper class (95% CI = [−0.4, 1.6] h/d, p = 0.1, n = 11). Steps per weekend day were also significantly lower compared to steps per weekday (mean of 14,060 steps vs. 15,860 steps, estimated location shift 1,850 steps, 95% CI  = [900, 2780] steps, p = 0.0002).

#### Level of PA and weight status

Direct accelerometry did not reveal significant differences between the amount of PA in overweight/obese children (median 4.7 h/d, IQR = [3.4, 6.4] h/d, mean 4.8 h/d, SD = 2.1) and the remaining peers (median 4.2 h/d, IQR = [3.1, 5.4] h/d, mean 4.4 h/d, SD = 1.8). The estimated location shift is 0.4 h/d, 95% CI = [−0.3, 1.2] h/d, p = 0.3.

#### Parental questionnaires

Parents were asked how many days per week their children were physically active for at least 60 minutes. A total of 79 parents answered “almost daily.” A comparison of PA from the “almost daily” group to the rest does not indicate any interrelation between the two (estimated location shift = 0.1 h/d, 95% CI = [−0.6, 0.7] h/d, p = 0.9): Only 66 of 95 children whose parents answered the pertinent questions participated in sports regularly, whereas 13 children never participated in sports, and 16 children participated in sports irregularly.

#### Preschool teachers' questionnaires

A total of 87 preschool teachers' questionnaires were evaluated. The evaluation of physical performance by preschool teachers was significantly correlated to the data obtained by direct accelerometry: Children who were evaluated as spending more time running around outside at the preschool also had higher values for PA using direct accelerometry (r = 0.28; p = 0.01). The correlation with the number of steps taken was just shy of being significant (r = 0.20, p = 0.06). Children who were evaluated as spending more time engaged in quiet activities (e.g. handicraft work or painting) took significantly fewer steps per day as measured by direct accelerometry (r = −0.28; p = 0.009), but did not show a significant correlation to PA (r = −0.13, p = 0.2). According to the evaluation completed by preschool teachers, boys participated significantly more often in leisure-time activities offered by preschools than girls did (p = 0.003). Boys were also evaluated as being more physically active than girls (p = 0.0005) and spending more time at outdoor activities (p = 5×10^−6^). By contrast, girls were reported to show significantly better performance in quiet activities than boys (p = 0.03) and had more balance between “playing centering on physical activity” vs. “arts and crafts and coloring” (p = 10^−6^). Like preschool teachers, parents also tended to evaluate boys to be more physically active than girls (p = 0.03).

Preschool teachers evaluated heavier children as being less physically active: With increasing BMI, children were judged to spend less time participating in “active playtime” (r = −0.22, p = 0.05), and to have parents who do not provide sufficient opportunity for running around (r = −0.28, p = 0.01). The preschool teachers reported that heavier children prefer games that do not involve physical activity (r = −0.23, p = 0.03). There is not a significant correlation between time spent in outdoor activities and weight status (r = −0.17, p = 0.1).

#### Influence of parental weight status on PA of children

The daily amount of PA in children with normal-weight parents (n = 23) was somewhat higher than that of children with at least one ov/ob parent (n = 44) (median 4.6 hours vs. 4.1 hours, though not significantly different (estimated location shift 0.6 hours, 95% CI = [−0.1, 1.1] hours, p = 0.1).

#### Media consumption and correlation to daily PA

**Figure 3:**
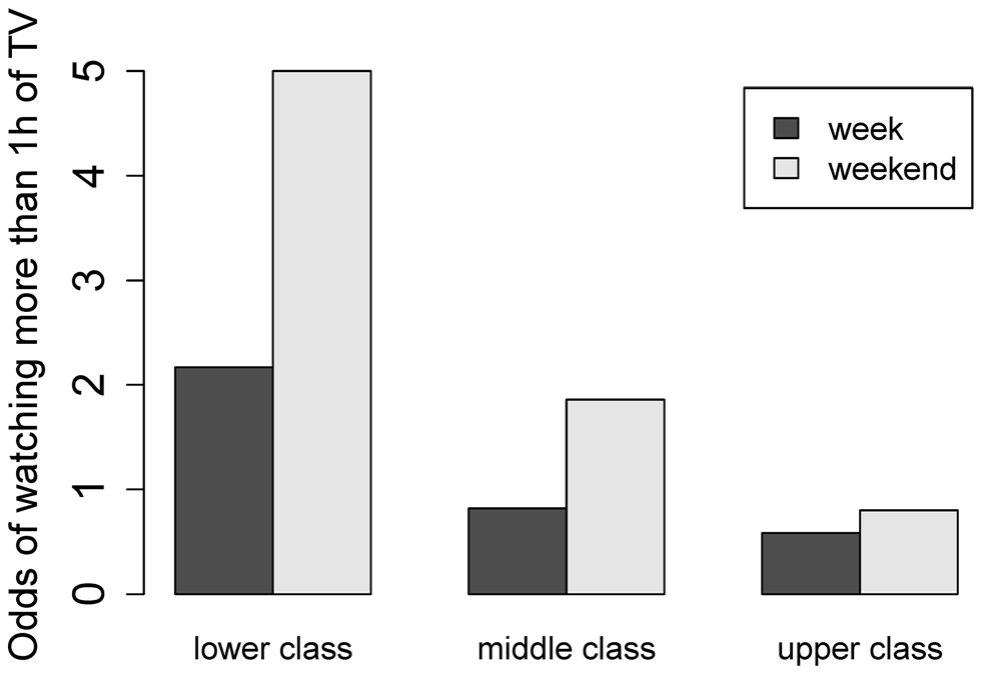
Odds ratio for TV consumption in preschool children aged 3–6 years for more than one hour on weekends and weekdays depending on social class (completed questionnaires on socioeconomic data were available from 78 families): The proportion of children who watch more than 1 hour of TV daily was significantly higher on the weekend compared to weekdays (p = 0.005) and increased with decreasing social class, both on weekdays (p = 0.05) and during the weekend (p = 0.01).

Daily hours of media consumption and computer playtime were evaluated by parental questionnaires, with data evaluated separately for weekdays and weekends. Media consumption was higher during the weekend. The proportion of children who watch more than 1 hour of TV daily was significantly higher on the weekend compared to weekdays (p = 0.005) and increased with decreasing social class, both on weekdays (p = 0.05) and during the weekend (p = 0.01) (Fig. 3). However, there was no significant correlation between daily media consumption and the amount of daily PA, neither on weekdays nor on weekends (week: r = 0.10, p = 0.4, weekend: r = −0.01, p = 0.9); nor between PA and playtime on the computer (week: r = −0.11; p = 0.3; weekend: r = 0.00; p = 1.0).

#### Predictive value of several parameters on BMI-SDS in preschool children

To test potential multivariate effects on childrens' weight status, a linear model was fit minimizing the sum of squares and where Akaike's information criterion as well as F-tests were used to assess the importance of each variable. Terms were dropped and added successively to optimize the AIC. The full linear model that was considered here took BMI-SDS as the independent variable and gender, age, amount of physical activity, amount of vigorous activity (> 6 MET), father's BMI, mother's BMI, the mother's age and socioeconomic status as explanatory variables. Akaikés information criterion suggested that only SES yields a considerable positive contribution whereby the mother's age and father's BMI were slightly informative, but not significant in a reduced linear model containing only these terms. This analysis was corroborated by an ANOVA for the model in which an F-test produced a p-value of 0.01 for SES, but in which each of the others p-values was above 0.3.

## Discussion

The present study has analyzed objectively measured PA data from 92 children aged 3–6 years attending German preschools over a median measuring period of 7 consecutive days and with a median measuring time of 23.5 h/d. Results reveal a significant difference between PA levels during the weekend (3.7 h/d) compared to weekdays (4.5 h/d) in children of this age group.

Averaged median daily PA was 4.3 hours/d (MET>3), and boys were consistently more physically active than girls when performing vigorous activities (MET>6). Objectively measured PA levels were neither related to weight status nor to the number of hours of daily media/computer consumption in this age group.

The suggested minimum amount of 60 minutes per day for this age group spent at moderate-to-vigorous physical activity (MVPA) was achieved by all children participating in our study when averaging their weekly values. All participating children were recruited from preschools from the Leipzig region, were 95% of 3–6 year old children attend preschools [27]. Thus, we may assume that data obtained from our study cohort resemble the “typical” PA behavior of children of this age group from this region. Several groups have assessed accelerometer-acquired estimates of MVPA in preschool-aged children, and values include 39.5±22.5 min/day (Puyau et al.), 46.8±27.6 min/d (Sirard et al.), 102.2±40.6 (Pate et al.) and 269.0±70.8 min/d (Freedson et al.) [28]. Due to variations in accelerometer cut points for activity levels among available studies regarding PA levels in preschool children, comparisons of accelerometer-derived MVPA estimates across studies remains a challenge [12], [28], [29].

Several studies have investigated objectively measured PA in children older than 6 years: A decline in PA, starting at school age (6–9 years), has been described by several groups [3], [4], [15], [25], [30], and PA levels measured by direct accelerometry have been reported to be lower in adolescents compared to school-aged children [31]. Interestingly, PA levels are still appropriately high in children at preschool age, matching or exceeding the recommendations of the scientific associations. Our results confirm and extend these previous observations by showing that PA levels in children aged 3–6 years are appropriately high, but that vigorous PA (MET>6) is already significantly higher in boys than in girls in this age group, although total physical activity (MET>3) is not different between genders. We also show that there are considerable differences in PA levels between weekdays and weekend days. In interpreting these results, cultural aspects in the former East Germany have to be considered: The vast majority of children in the investigated group attend a preschool. Generally, this involves play time outdoors and significant amounts of running around. Our results demonstrate that this level of activity is not maintained by parents on the weekends in general. On the weekend, the children are also likely to be spending less time with outdoor activities with other children of the same age.

The phenomenon of higher PA levels on weekdays compared to weekends has been previously reported for children older than 6 years, but most of them utilized accelerometer data from≤4 days [3], [30], [32]. Only one study has previously reported a similar pattern of weekday and weekend day variability in preschool-aged children, however, mean duration of monitoring was only 12.5 h/d [33]. Thus, to the best of our knowledge, this is the first study to report differences in objectively measured PA levels over the course of the week in preschool-aged children over a period of 7 consecutive days with a median measuring time of 23.5 hours.

Studies that have investigated PA levels in children younger than 6 years, considering weight status and socioeconomic factors, are limited [12], [34]. SES did not seem to be a significant factor in explaining the amount of time spent in PA or sedentary behavior [34]. However, although SES does not seem to be a predictor of daily physical activity of children in that age group, we show that SES seems to influence weight status already in preschoolers. A recent meta-analysis that has evaluated 29 articles representing 6,309 children aged 3–5 years has shown that children of this age engaged on average in 42.8 min moderate-to-vigorous physical activity, 54.4 min for boys and 45.4 min for girls [12]. Although children of our study group had higher daily values for moderate-to-vigorous physical activity, our results confirm the previous findings of boys in this age group being more physically active than girls [12], [16].

There is still no “gold standard” in defining the different activity levels in childhood and there are considerable variations in accelerometer cut points for activity levels among available studies regarding PA in preschool-aged children [12], [28], [29], [35], [36]. Thus, we had to choose one system based on validated data available in the literature to define the activity levels in our study cohort. We are aware of the fact that this choice can affect results significantly, but we choose values that were established and validated for this age group by Pate et al. and that are widely used by other groups [16], [23]. To validate this model in more detail, we have compared the total energy expenditure estimated using the SenseWear Pro® armband with values published by Reilly et al [37]. The results suggest that the assigned values for METs are reasonably good, but that there is likely even more moderate physical activity than we reported, based on the definitions available in the literature. However, total energy expenditure of our study cohort is comparable to that published by Reilly's group [37]. Since we have analyzed a total of 834,000 minutes, one of the main findings of our study, namely the differences in activity patterns between weekdays and weekend days, provide a robust analysis of physical activity patterns during the course of the week and does not seem to be influenced by the chosen cut points or model for activity levels in this age group.

A sub-analysis of our study has compared PA levels in normal-weight and overweight/obese children. Prevalence rates for overweight and obesity in our study population are in accordance with data from the national KIGGS survey [25] and a recent national evaluation [5]. Our results confirm previous findings, as the weight of the children was affected by social status already in this age group, with more overweight/obese children living in families of low social status. Maternal weight status seems to have a significant impact on the weight status of their children. However, this association was not statistically significant in our study.

Our results revealed no significant differences in PA levels among normal-weight and overweight/obese children in this age group. Similar data were presented in a Portuguese study with children of equivalent ages [32]. In contrast, one study in children of similar age showed that age, gender, race, and BMI z-score were correlated to moderate-to-vigorous physical activity in this age group, and several studies performed in older children (>6 years) showed a close and significant relationship between weight status and level of physical activity, with overweight and obese children being significantly less physically active than their normal-weight peers [38]–[40]. Extending these findings in older children, a recent study revealed a close relationship between percent body fat and the level of physical activity, measured as steps per day and moderate and vigorous physical activity in both genders of children aged 8, 10 and 12 years old [41]. The fact that we did not find a correlation between weight status and level of PA in children of this age group leads us to the conclusion that in younger children the natural drive to move is not influenced yet by higher body weight, as it seems to be in older children. Therefore, it is essential to start preventive interventions already at this age. The urgent need for early intervention is underlined by the fact that PA levels seem to decrease from childhood to adolescence, as discussed above. As there are no validated obesity prevention strategies in childhood and adolescence, early identification of potential risk factors are crucial. In adults, the physical activity status of an individual may predict the risk for developing chronic diseases in the future. However, our results suggest that risk prediction for obesity and associated diseases may not be assumed by the level of physical activity in preschool-aged children, but physical activity patterns may be of increasing importance during the course of childhood development [42]. Thus, promotion of physical activity that starts as early in life as possibly may prevent chronic diseases later in life.

Additional variables that seem to have an impact on the child's PA level include parental level of PA, preschool quality, home PA equipment, and child's enjoyment of PA. Whereas there is a significant relationship between the family support, quality of preschool attended, home PA equipment, child's enjoyment of PA and the daily amount of the child's PA, there does not seem to be a direct relationship between parent's and the child's level of physical activity [43]. In addition, the amount of daily media consumption is assumed to influence physical activity in children [44]. However, our data showed no significant correlation between the hours of daily media consumption and number of hours of daily physical exercise, which is in accordance with a previous study, showing that increased TV viewing is not associated with lower total energy expenditure in children [45]. In our study, TV consumption and computer playtime (including gameboy and playstation) of participating children were evaluated, both, during weekdays and on the weekend, based on parental questionnaires. However, additional factors that might influence sedentary behavior in this age group, such as books, drawing and coloring, playing board games and listening to stories on CD, were not addressed in the parental questionnaires. Neither TV consumption nor playtime on the computer was related to the amount of daily physical activity. The potential effects of the above mentioned additional factors of sedentary behavior, which have not been addressed by the applied questionnaires, cannot be ruled out. On the other hand, media consumption provides only one aspect of an individual's sedentary behavior habits, which are potentially correlated to a variety of factors, including the number of siblings living in the home, parental enjoyment of PA, child's enjoyment of PA, child's athletic coordination compared with peers, distance to the nearest park, and others [46]. These factors have not been considered in the present study, which might as well – at least in part – explain the missing relationship between PA and media consumption in our study cohort.

### Strengths of this study

To the best of our knowledge, this is the first study that has analyzed objectively measured PA levels using SenseWearPro® accelerometers in a cohort of 92 children aged 3–6 years over a median measuring period of 7 consecutive days, including one day of the weekend, with a median measuring time of 23.5 h/d. We did not find comparable studies in the literature that continuously examined such a large cohort of children of comparable age over such a long period of time, providing a total of 834,000 analyzed minutes of accelerometry. A 4–7-day monitoring protocol allows reliable estimates of PA behavior and activity patterns [47]. This study combined 3 different sources to consolidate the activity data of children: direct accelerometer data, data from parental questionnaires, and data from preschool teachers' questionnaires and included sub-analyses taking into consideration the weight status and the socioeconomic status of the family.

### Limitations of this study

The participation rate of the recruited preschoolers was between 6% and 70% and seemed to be influenced by the social status of the screened families and by the commitment of preschool teachers. A limiting factor for the value of the collected questionnaire data might have been the subjective perception of the parents regarding the physical activity of their children. In addition, the fact the preschool teachers spend more time with the children over the course of the week than parents do might have led to some bias in terms of evaluating “real activity levels” of participating children. Another aspect that might have influenced the quality of the data was the duration of the recruiting period (i.e. spanning all seasons), which might have led to a potential seasonal bias on the obtained data, though an analysis of this data did not reveal one. Supporting this, a study on seasonality and preschoolers' PA engagement revealed that differences in PA across seasons were much less evident during the time the children attended preschools [48]. An additional limitation might arise from the point that variations in accelerometer cut points for activity levels among available studies regarding PA in preschool children may result in disparate estimates of PA levels and may bias comparisons across studies, as discussed in more detailed above [12], [29].

### Summary and conclusions

The level of PA in children aged 3–6 years was measured by direct accelerometry over a median of 7 consecutive days. Median daily PA (MET>3) was 4.3 hours, and the suggested minimum amount of PA for this age group of 60 min/d spent at moderate-to-vigorous physical activity was achieved by every child when its values for the week were averaged. Boys were more vigorously active than girls. Daily PA was higher during weekdays compared to weekends in this age group. No external factors or environmental conditions that are known to affect PA of older children, such as BMI or media consumption, influenced activity behavior in preschool-aged children. Our data demonstrate that preschool-aged children seem to be active enough, as suggested by the scientific communities, independent of weight status and TV consumption. However, studies of older children do indicate associations between weight status and both physical activity and TV consumption. Presumably habits are formed at this early age. It may not be as important then to increase the amount of physical activity in preschool-aged children participate in as it is to foster the awareness of its importance and turn it into a regular part of daily habit especially on the weekends. Long-term follow-up studies are necessary to further investigate such questions.
